# DDGun: an untrained predictor of protein stability changes upon amino acid variants

**DOI:** 10.1093/nar/gkac325

**Published:** 2022-05-07

**Authors:** Ludovica Montanucci, Emidio Capriotti, Giovanni Birolo, Silvia Benevenuta, Corrado Pancotti, Dennis Lal, Piero Fariselli

**Affiliations:** Genomic Medicine Institute, Lerner Research Institute, Cleveland Clinic, 9500 Euclid Avenue, Cleveland, OH 44195, USA; BioFolD Unit, Department of Pharmacy and Biotechnology (FaBiT), University of Bologna, Via F. Selmi 3, 40126 Bologna, Italy; Department of Medical Sciences, University of Torino, Via Santena 19, 10126, Torino, Italy; Department of Medical Sciences, University of Torino, Via Santena 19, 10126, Torino, Italy; Department of Medical Sciences, University of Torino, Via Santena 19, 10126, Torino, Italy; Genomic Medicine Institute, Lerner Research Institute, Cleveland Clinic, 9500 Euclid Avenue, Cleveland, OH 44195, USA; Department of Medical Sciences, University of Torino, Via Santena 19, 10126, Torino, Italy

## Abstract

Estimating the functional effect of single amino acid variants in proteins is fundamental for predicting the change in the thermodynamic stability, measured as the difference in the Gibbs free energy of unfolding, between the wild-type and the variant protein (ΔΔ*G*). Here, we present the web-server of the DDGun method, which was previously developed for the ΔΔ*G* prediction upon amino acid variants. DDGun is an untrained method based on basic features derived from evolutionary information. It is antisymmetric, as it predicts opposite ΔΔ*G* values for direct (A → B) and reverse (B → A) single and multiple site variants. DDGun is available in two versions, one based on only sequence information and the other one based on sequence and structure information. Despite being untrained, DDGun reaches prediction performances comparable to those of trained methods. Here we make DDGun available as a web server. For the web server version, we updated the protein sequence database used for the computation of the evolutionary features, and we compiled two new data sets of protein variants to do a blind test of its performances. On these blind data sets of single and multiple site variants, DDGun confirms its prediction performance, reaching an average correlation coefficient between experimental and predicted ΔΔ*G* of 0.45 and 0.49 for the sequence-based and structure-based versions, respectively. Besides being used for the prediction of ΔΔ*G*, we suggest that DDGun should be adopted as a benchmark method to assess the predictive capabilities of newly developed methods. Releasing DDGun as a web-server, stand-alone program and docker image will facilitate the necessary process of method comparison to improve ΔΔ*G* prediction.

## Graphical Abstract

**Graphical Abstract:**
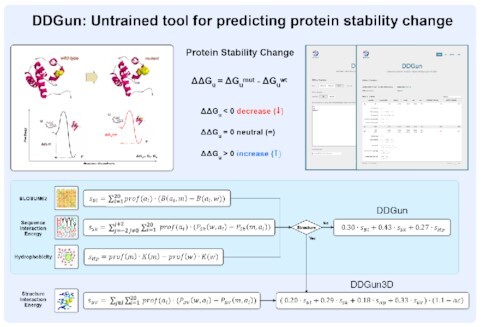
DDGun: an untrained predictor of protein stability changes upon amino acid variants.

## INTRODUCTION

Predicting the change in protein stability upon single amino acid variants constitutes a crucial step toward understanding the relationship between protein structure and function. Elucidating this relationship will deepen our knowledge about the biophysics of protein folding and will provide a tool to decipher genomic variation in the light of biological function and molecular mechanisms of health and disease (1–3), directly guiding clinical applications toward personalized treatments.

The impact on the protein stability of the substitution of a single amino acid is measured through the ΔΔ*G*, which is the difference in the free energy of unfolding (Δ*G*) between the wild-type and the variant protein: ΔΔ*G* = Δ*G*_variant_ – Δ*G*_wild-type_. Several methods, based on either sequence (4,5) or sequence and structure information (5–8), have been developed for the prediction of ΔΔ*G* upon single residue variation (9–11) and two methods are also applicable to multiple-residue variants (12,13). Prediction performances reach correlation coefficients ranging from 0.4 to 0.6 for single-site variants. ΔΔ*G* prediction is therefore not fully resolved due to several limitations and challenges (14), and further work is required to bring ΔΔ*G* prediction methods to accuracies suited for biophysical and clinical applications. The main limitations due to the characteristics of the available data sets are: their intrinsic uncertainty and distributions which limits prediction accuracies (15) and the bias of common experimental dataset toward destabilizing variants (14). Major challenges concerning methods design are: avoiding overfitting due to similarity between the sequences of the training and testing dataset, and developing a method which fulfil the anti-symmetrical property by predicting opposite ΔΔG values for direct (A → B) and reverse (B → A) variants. Although the biophysics of the folding process imposes the anti-symmetricity of the ΔΔ*G* for reverse variants, most available methods lack this property (16). Due to all these challenges, a robust comparison among these predictors is a difficult task. Thus, it is essential to derive new curated data sets and to derive benchmark methods.

Here we present the web server for the DDGun method (17) which was developed as a non-trained method based on simple anti-symmetrical features, hence addressing anti-symmetry and avoiding overfitting as it is an untrained method. Initially developed as a baseline-benchmarking tool, DDGun reaches prediction performances comparable to trained methods and constitutes a valid alternative tool as recently shown (18).

## MATERIALS AND METHODS

### Sequence-based DDGun

#### Evolutionary scores

**Figure 1.**
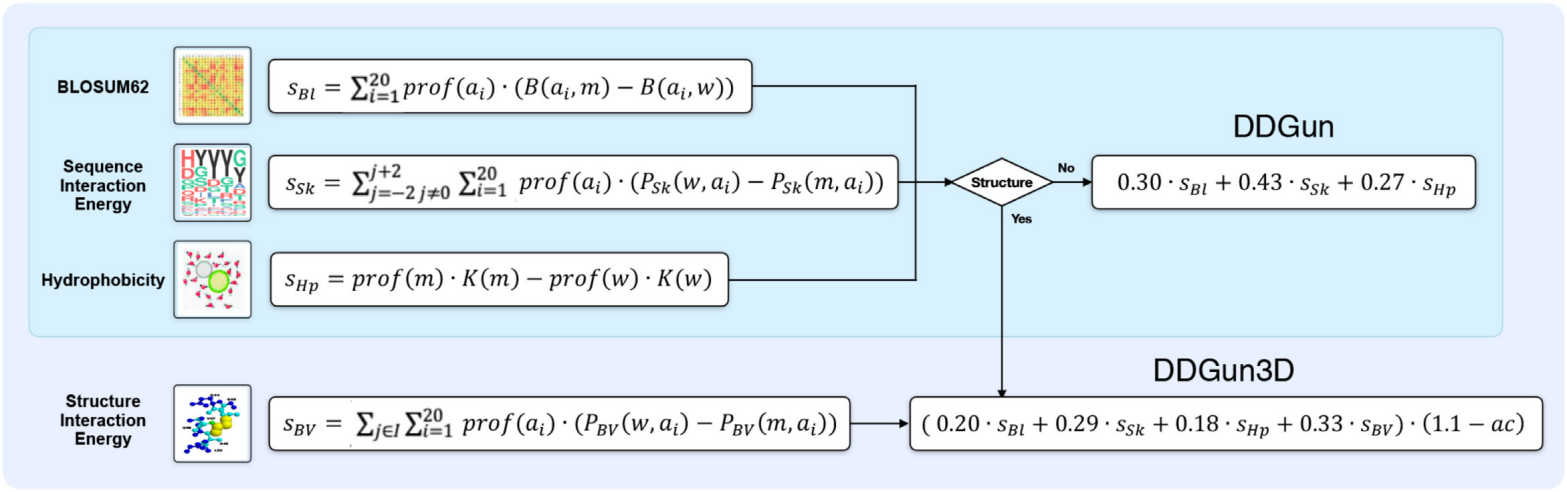
Method overview: building DDGun and DDGun3D through the individual scores. In all the equations, *m* indicates the variant residue, *w* indicates the wild-type residue and *prof(a_ij_)* indicates the profile, that is frequency of *i*-th amino acid (*a_i_*) at the *j*-th position of the multiple sequence alignment. *B(a_i_,a_j_)* is the substitution score given by the BLOSUM62 matrix between the *i*-th and *j*-th amino acids. *P_Sk_(a_i_,a_j_)* is the Skolnick interaction potential between residues *i*-th and *j*-th; *K(a)* is the hydrophobicity of amino acid *a* as measured by the Kyte-Doolittle scale*; P_BV_(a_i_,a_j_)* is the Bastolla-Vendruscolo pairwise statistical potential between residues *i*-th and *j*-th. In the linear combination of DDGun3D, *ac* is the relative solvent accessibility of the residue.

DDGun predicts the ΔΔ*G* through a linear combination of scores based on evolutionary information. These scores are summarized in Figure 1 and are: (i) the difference between the wild-type and variant residue in the BLOSUM62 substitution matrix (19) (*s_Bl_*), which takes into account the difference in evolutionary conservation between the wild-type and variant residue; (ii) the difference in the interaction energy—measured through the Skolnick statistical potential (20)—between the wild-type and variant residue within a 2-residue-long sequence window (*s_Sk_*), which takes into account the difference between wild-type and variant residues in the interaction energy with their near neighbours in the sequence; (iii) the difference in the hydrophobicity between wild-type and mutant residues according to the Kyte-Doolittle scale (*s_Hp_*).

For each of these scores, the differences between the wild-type and variant amino acids in the three features (evolutionary conservation, interaction energy and hydrophobicity) are computed as summation over all the possible amino acids, weighted through the sequence profile derived from the multiple sequence alignments (equations in Figure 1).

Interestingly, the evolutionary scores defined as such, are anti-symmetric by design. They take, by construction, opposite values for direct and corresponding reverse variants (being the direct variant substituting residue A with residue B, and the reverse being substituting back residue B with residue A); therefore, the method is anti-symmetrical even without training on direct and inverse variants.

#### Scores combination

These three sequence-based evolutionary scores were then combined through a simple linear combination to build the DDGun predictor of ΔΔ*G*. The linear combination weights were chosen to be proportional to the ΔΔ*G* values available in the high quality and manually curated VariBench data set (21) and normalized to 1. The weights are: 0.30, 0.43 and 0.27 for *s_Bl_*, *s_Sk_* and *s_Hp_*, respectively. The linear combination of DDGun is shown in Figure 1. It has to be stressed that the weights of the linear combinations were not chosen to fit the ΔΔ*G* prediction in any data set, making DDGun a fully untrained method.

### Structure-based DDGun

#### Structure-based scores

The structure-based version of DDGun (DDGun3D) is based on the three previously defined sequence-based scores and one additional structure-based score that considers the variation of the structural environment. In order to compute this score, a 3D protein structure must be resolved. This additional score, *s_BV_*, takes into account the differences in the interaction energy between the wild-type and variant residue with their structural environments, defined as a sphere of radius 5 Å centered in the variant site. The interaction energy between wild-type and variant residues with their structural environments is calculated through the Bastolla-Vendruscolo statistical potential (22). As before, the difference in the interaction energy is weighted over the sequence profile (*s_BV_* equation in Figure 1). It is worth noticing that the structural environment is computed on only the wild-type structure and therefore does not consider possible structural rearrangements that may occur upon mutation, and this may be a source of a partial antisymmetry loss.

#### Scores combination

Structure based DDGun predictions are given by a linear combination of the four scores shown in Figure 1 (three of which are sequence-based and the last one is structure-based). Also, for the structure-based version of the method, the scores were combined through a linear combination whose weights were chosen to be proportional to the ΔΔ*G* values available in the VariBench data set normalized to 1. The weights are: 0.20, 0.29, 0.18 and 0.33 for *s_Bl_*, *s_Sk_*, *s_Hp_* and *s_BV_*, respectively. For the structural method, an additional modulation factor has been introduced to take into account that variants at solvent-exposed sites tend to have lower effects on the ΔΔ*G* values. This factor is (1.1 - *ac*) where *ac* is the relative solvent accessibility of the residue computed through the DSSP program (22,23). As a first approximation, the modulation factor was considered linear, even though the effect of accessibility could be more complex. When the solvent accessibility is small or 0 (i.e. buried residues) the modulation factor is 1 (or almost 1) and hence the predicted ΔΔ*G* is maximum. When solvent accessibility is high (exposed residues), the modulator factor approaches 0 and hence the predicted ΔΔ*G* is reduced. Therefore, DDGun tends to predict higher ΔΔ*G* changes for buried residues and smaller ΔΔ*G* changes for exposed residues. The equation for the linear combination with the modulation factor is shown in Figure 1. Structure-based score and accessibility were computed on the single chain.

### DDGun for multiple variants

This method is designed in a way that is easily extended to predict ΔΔ*G* upon multiple site variants. Among the methods which provide this option, DDGun is the only sequence-based one. Given multiple site variants, DDGun predicts the ΔΔ*G* changes for each variant separately and then it combines the prediction using the following equation:

$$\begin{equation*}{s_{{\rm mult}}} = {\rm max}\left( {{s_s}} \right)\ + {\rm min}\left( {{s_s}} \right)\ - {\rm mean}\left( {{s_s}} \right)\end{equation*}$$

where ${s_{{\rm mult}}}$ is the ΔΔ*G* prediction for the multiple site variant, and ${s_s}$ is the vector ${s_s} = ( {{s_1},{s_2},\ \ldots {s_M}} )$ with the ΔΔ*G* predictions for each variant separately through DDGun. This formula derives from the hypothesis that the minimum and the maximum values are likely to be the most relevant in affecting the ΔΔ*G* resulting from a multiple site variant. Thus, the final score has been defined as the sum of the minimum and maximum values of the ΔΔ*G* predictions for the single variants and this sum was then centered around the mean of the ΔΔ*G* predictions for all the simultaneous variants (by subtracting the average of the ΔΔ*G* prediction).

### DDGun update

While the equations and score weights are the same as those of the original version of DDGun, the web-server version has been updated in the protein sequence database against which the multiple sequence alignments are computed. Indeed, a critical factor for DDGun, shared with all evolutionary-based methods, is the sequence profile, which is derived from the multiple sequence alignment and modulates every score computed by DDGun (see Figure 1). The sequence profile is highly dependent on the quality of the multiple sequence alignment on which it is based, being more accurate the bigger it is the number of aligned sequences. The multiple sequence alignments of the original DDGun method were obtained aligning each protein against the uniprot2016 database (February 2016) through the *hhblits* program (23). We have updated the method for the web server version by replacing Uniprot2016 with a newer release, Uniclust30 (August 2018) and by running *hhblits* with an *e*-value of 10^–6^. It has to be noted that the weights of the linear combinations were not recomputed with the new multiple sequence alignments, and are the same reported in the original DDGun method.

## RESULTS

### Method performances

#### Testing data sets

To evaluate the performances of DDGun, we report the Pearson correlation coefficients (*r*) and the root mean square error (RMSE) on two new data sets which were not available when DDGun was developed. The first one, s96, is a data set of single site variants which consists of 96 variants from 14 proteins derived from the latest version of ProTherm released in 2021 (24). The variants were manually checked and corrected according to the information derived from the papers reporting the experimental values. Only variants on proteins having less than 25% sequence identities with proteins in S2648 (25) and VariBench (21) were selected. This data set is hence complementary to S2648 and VariBench and represents a blind test for all the methods trained on S2648 or VariBench, as well as a valid test for generalization properties for all methods. The second dataset m28 is a dataset of multiple site variants derived from the same latest version of ProTherm (released in 2021). Only variants for which ΔΔ*G* or ΔΔ*G*_H2O_ were reported after 2013, hence not included in the previous data sets, were retained. In both data sets, s96 and m28, when multiple experimental ΔΔ*G* values were reported for the same variant, the average has been taken. A description of these new data sets is reported in Supplementary Table S1 and the full data sets are available as supplementary material and on the DDGun web server.

#### DDGun performances

**Figure 2.**
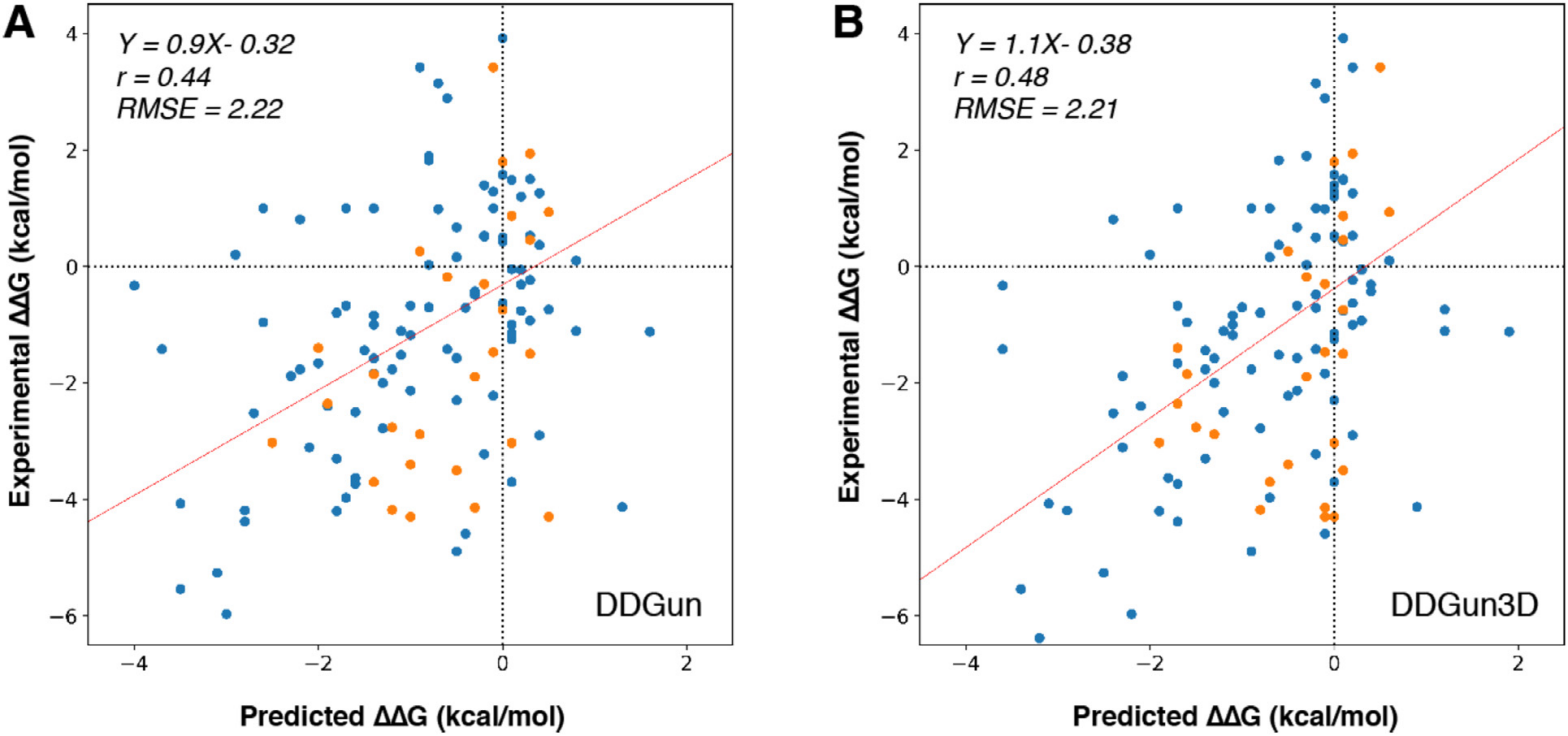
Scatter plot of predicted and experimental ΔΔ*G* on s96 (blue) and m28 (orange) datasets for DDGun (**A**) and DDGun3D (**B**). RMSE and *r* are defined in supplementary materials.

We first re-computed the performances of the web server version of DDGun, which rely on an updated version of the Uniclust database, on the old datasets. On the largest available data sets (VariBench and S2648 with 1432 and 2648 variants, respectively), the performances of DDGun reach a correlation of 0.48 and 0.49 and those of DDGun3D reach 0.54 and 0.57 for each dataset, respectively. The performances are similar (average difference 1–2%) to those obtained with the original DDGun version. Similar tests were performed to evaluate the performance of DDGun Ssym (26), an anti-symmetric data set of single point mutations, and PTmul (17) which collects multiple site variations. Also, in this case the performance of the newer version of DDGun is consistent with the previous one. The scoring indices of DDGun on these data sets are reported in Supplementary Table S2 and S3. On the new datasets, DDGun and DDGun3D performances are shown in Table 1. On s96, DDGun reaches a correlation coefficient of 0.48 and DDGun3D reaches a correlation coefficient of 0.52 between experimental and predicted ΔΔ*G* values. Root mean square errors are 2.14 and 2.10 kcal/mol, respectively. Hence, on a data set of single point variants which is complementary to those used for the development of the methods, DDGun reaches roughly the same performance. On multiple variants, on the new data set m28 DDGun and DDGun3D reach a correlation of 0.42 and 0.44 with root mean square errors of 2.49 and 2.54 kcal/mol, respectively (Table 1). Merging both data sets (s96 and m28) DDGun and DDGun3D reach a correlation of 0.44 and 0.48, respectively, and with a root mean square error of 2.2 kcal/mol (Figure 2).

**Table 1.** Performances of DDGun on the two new datasets.

	s96		m28	
Method	*r*	RMSE	*r*	RMSE
DDGun	0.48	2.14	0.42	2.49
DDGun3D	0.52	2.10	0.44	2.54
FoldX	0.22	4.18	0.38	2.64
Maestro	0.36	2.29	0.28	2.90
INPS-MD	0.43	2.21	na	na
mCSM	0.31	2.33	na	na
INPS-Seq	0.44	2.20	na	na
PopMusic	0.36	2.30	na	na
SDM	0.51	2.12	na	na

*r*: Pearson's correlation coefficient between the predicted and experimental ΔΔ*G* values; RMSE: root mean square error (expressed in kcal/mol). Measures of performance are defined in Supplementary Data.

In order to compare DDGun performances, we also report in Table 1 the performances of other widely used methods on the s96 and, when applicable, on the m28 data set. The fact that the performances of DDGun are stable across different datasets, including new and blind ones, proves that DDGun represents a robust assessment of the predictive capabilities of the simple evolutionary and energetic features and constitutes a robust benchmark for more complex methods.

### Web-server description

**Figure 3.**
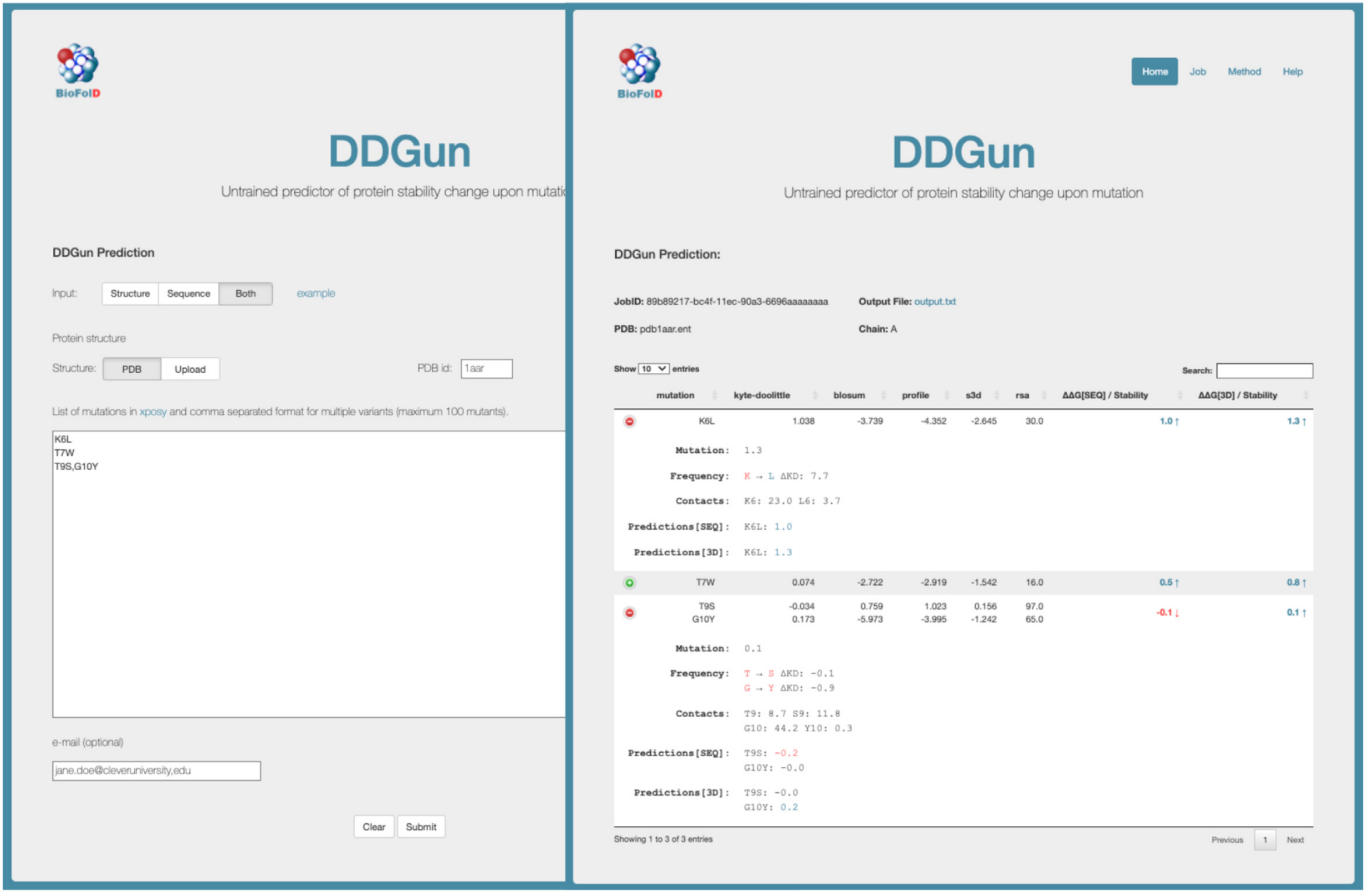
To make a ΔΔ*G* prediction on the server, the sequence or the structure-based inputs must be selected by clicking on the corresponding button. A third option (‘Both’) allows to run both predictions providing in input the structural information. The web interface of the sequence-based DDGun consists of two textarea boxes, the first one for the protein sequence (simple text or fasta format) and the second one for the list of variants (e.g. T7W). Multiple variants are accepted as comma-separated in the same line. The output of DDGun shows the predictions along with the individual scores. In the last columns, the prediction of the ΔΔ*G* of unfolding in kcal/mol is displayed, The impact of the variants on protein stability is represented with a blue upwards arrow (↑) indicating an increase of stability, or a red downward arrow (↓) indicating a decrease of stability. The equal sign ( = ) indicates variants with no effect on protein stability. A green plus button at the beginning of each row visualises further details associated with each prediction, such as the frequency of the wild-type and mutated residues in the variation site of the protein sequence profile and the interactions of the wild-type residue within a distance of 5 Å.

A graphical view of a web-server prediction is shown in Figure 3.

#### DDGun input

To make a ΔΔ*G* prediction with the DDGun server, the user should first choose whether to run the sequence-based, the structure-based or both versions of DDGun, by clicking on the corresponding ‘Sequence’, ‘Structure’ or ‘Both’ button. The web interface of the sequence-based DDGun consists of two textarea boxes, the first one for the protein sequence (simple text or fasta format) and the second one for the list of variants (e.g. K6L). Multiple variants are accepted and should be introduced in the same line, comma-separated. When running the structure-based method, it is possible to either upload a PDB file or insert the PDB code. When selecting the ‘Both’ option, the server requires the same input as that of the structure-based method, but it also returns the sequence-based predictions. The server internally processes the PDB file extracting the mutated protein chain keeping the original amino acid numbering, including the amino acids with identified insertion codes. If the option ‘Both’ is selected, the protein sequence is determined from the lists of resolved amino acids. On the server web page-specific links allow loading examples of possible inputs.

#### DDGun output

The output of DDGun is shown on a web page in tabular form. The results for a variant are shown in each line, along with the individual scores (three for the sequence-based version and four scores plus the relative solvent accessibility for the structure based one). In the last column, the prediction of the ΔΔ*G* of unfolding (ΔΔ*G*[SEQ] or ΔΔ*G*[3D]) is displayed, expressed in kcal/mol. The effect of the variant on protein stability is also represented by an upwards (↑) or downwards (↓) arrow corresponding to a stabilizing or destabilizing mutation, respectively. An equal sign ( = ) indicates the predicted variants with neural effect (ΔΔ*G* = 0.0 kcal/mol). A green plus button at the beginning of each row allows to visualise further details associated to each prediction, such as the frequency of the wild-type and mutated residues in the variation site of the protein sequence profile, and the interactions of the wild-type residue within a sphere of 5 Å. When the option ‘Both’ is selected, DDGun returns both ΔΔG[SEQ] and ΔΔG[3D], which are the predicted ΔΔ*G* based on only sequence information and on sequence and structure information, respectively. The component view makes the DDGun predictions explainable allowing a clearer interpretation of the method. The output is stored on the server for about one day, and it is accessible using the JobID provided at the beginning of the output page. If the user, upon query, provides the email, the output will also be sent by email as an attachment in text format.

## CONCLUSIONS

DDGun web server is the released version of the previously developed DDGun method, an untrained method for the prediction of ΔΔ*G* upon single and multiple site variants, which is based on simple anti-symmetrical conservation and energetic scores. DDGun was among the top-performing methods when benchmarked with other 21 tools on a new dataset (18).

In respect to the original DDGun method, the web server version relies on an updated version of the protein sequence database (Uniclust2018 instead of Uniprot2016). In this work, we show the performance of the updated version of DDGun on the main datasets of experimental ΔΔ*G* values and we also test it on two new datasets never seen before. We show that the performance is consistent across datasets and across DDGun versions, reaching prediction correlation comparable to the state of the art, despite DDGun being an untrained method. This qualifies DDGun as a robust benchmark method for ΔΔ*G* prediction and method comparison.

## DATA AVAILABILITY

DDGun is freely available as a web server at: https://folding.biofold.org/ddgun for interactive queries. A stand-alone version to run DDGun locally is available on GitHub: https://github.com/biofold/ddgun. We also provide a docker image hosted on DockerHub (https://hub.docker.com/repository/docker/biofold/ddgun) which allows reproducing the analysis identically as presented in this study. DDGun is freely accessible at http://folding.biofold.org/ddgun.

## Supplementary Material

gkac325_Supplemental_Files
