# Supplementary material for: DDGun: an untrained predictor of protein stability changes upon amino acid variants
Source: Nucleic Acids Res. 2022 May 7;50(W1):W222–7. doi: 10.1093/nar/gkac325 (PMC9252764; doi:10.1093/nar/gkac325)
Supplement: gkac325_Supplemental_Files [file gkac325_supplemental_files.zip › 20220415-ddgun-supmat.pdf]

SUPPLEMENTARY MATERIALS

DDGun: an untrained predictor of protein stability changes upon amino acid variants

*Ludovica Montanucci[1]†, Emidio Capriotti[2]†, Giovanni Birolo[3], Silvia Benevenuta[3], Corrado Pancotti[3], Dennis Lal[1] and Piero Fariselli[3]**

[1] Genomic Medicine Institute, Lerner Research Institute Cleveland Clinic, 9500 Euclid Avenue, Cleveland, OH 44195, USA.
[2] BioFolD Unit, Department of Pharmacy and Biotechnology (FaBiT), University of Bologna, Via F. Selmi 3, 40126 Bologna, Italy.
[3] Department of Medical Sciences, University of Torino, Via Santena 19, 10126, Torino, Italy.

* To whom correspondence should be addressed. Email: piero.fariselli@unito.it
† Joint Authors.

Performance measures

For evaluating the performance of the methods in the regression task we compared the predicted (*p*) and experimental (*e*) values of the variation of unfolding free energy change upon mutation (ΔΔG).

The standard scoring values calculated in our assessment are the Pearson correlation coefficients (*r*) and the root mean square error (RMSE). They are defined as follows:

$$r = \frac{\sum_{i=1}^{N}\left(\Delta\Delta G_e - \overline{\Delta\Delta G_e}\right)\left(\Delta\Delta G_p - \overline{\Delta\Delta G_p}\right)}{\sqrt{\sum_{i=1}^{N}\left(\Delta\Delta G_e - \overline{\Delta\Delta G_e}\right)^2}\ \sqrt{\sum_{i=1}^{N}\left(\Delta\Delta G_p - \overline{\Delta\Delta G_p}\right)^2}} \quad [1]$$

$$RMSE = \sqrt{\frac{\sum_{i=1}^{N}(\Delta\Delta G_p - \Delta\Delta G_e)^2}{N}} \quad [2]$$

where $\underline{\Delta\Delta G_p}$ and $\underline{\Delta\Delta G_e}$ are the average predicted and experimental ΔΔG values respectively. In the case of antisymmetric datasets (Ssym) we calculated the Pearson correlation coefficient between direct and inverse predictions ($r_{dir\text{-}inv}$) and the average bias $\langle\delta\rangle$ as follows:

$$r_{dir-inv} = \frac{\sum_{i=1}^{N}\left(\Delta\Delta G_p^{inv} - \overline{\Delta\Delta G_p^{inv}}\right)\left(\Delta\Delta G_p^{dir} - \overline{\Delta\Delta G_p^{dir}}\right)}{\sqrt{\sum_{i=1}^{N}\left(\Delta\Delta G_p^{inv} - \overline{\Delta\Delta G_p^{inv}}\right)^2}\ \sqrt{\sum_{i=1}^{N}\left(\Delta\Delta G_p^{dir} - \overline{\Delta\Delta G_p^{dir}}\right)^2}} \quad [3]$$

$$\langle\delta\rangle = \frac{\sum_{i=1}^{N}(\Delta\Delta G_p^{dir} - \Delta\Delta G_p^{inv})}{N} \quad [4]$$

According to Equations 3 and 4, a perfect anti-symmetric method would yield $r_{dir\text{-}inv}$ value of -1 and $\langle\delta\rangle$ of 0 kcal/mol.

Table S1. Composition of the data sets of experimental ΔΔG.

Dataset	PDBs	Mutants	Stabilizing	Destabilizing
VariBench	79	1,432	388 (27.1%)	1044 (72.9%)
S2648	132	2,648	602 (22.7%)	2,046 (77.3%)
Ssym	357	684	342 (50.0%)	342 (50.0%)
s669	96	669	170 (25.4%)	499 (74.6%)
PTmul*	89	858	259 (30.2%)	599 (69.8%)
s96	14	96	32 (33.3%)	64 (66.7%)
m28	12	28	7 (25.0%)	21 (75.0%)

Stabilizing variants are those with unfolding ΔΔG≥0. * 1PGA mutations were excluded because the sequence profile is composed of a small number of sequences (≤10).

Tab. S2. Performances of DDGun and DDGun3D web server version on previously collected data sets

Method	VariBench		S2648		S669	
	r	RMSE	r	RMSE	r	RMSE
DDGun	0.48	1.73	0.49	1.44	0.39	1.71
DDGun3D	0.54	1.70	0.57	1.34	0.42	1.59

r: is the Pearson's correlation coefficient between the predicted and experimental ΔΔG values; RMSE: root mean square error (expressed in kcal/mol); Measures of performance are defined above.

Tab. S3. Performances of DDGun and DDGun3D web server on balanced (Ssym) and multiple-variant datasets

**Method**	**Ssym**						**PTmul***	
	r_{dir}	$RMSE_{dir}$	r_{inv}	$RMSE_{inv}$	$r_{dir\text{-}inv}$	$\langle\delta\rangle$	r	RMSE
DDGun	0.46	1.49	0.45	1.51	-0.99	-0.05	0.35	2.22
DDGun3D	0.55	1.42	0.52	1.46	-0.99	-0.05	0.38	2.24

r: is the Pearson's correlation coefficient between the predicted and experimental ΔΔG values; RMSE: root mean square error (expressed in kcal/mol); $r_{dir\text{-}inv}$ is the Pearson correlation between direct and inverse variants. $\langle\delta\rangle$ is the average bias and is expressed in kcal/mol. Measures of performance are defined above. *1PGA mutations were excluded because the sequence profile is composed of a small number of sequences (≤10).
